# First-Principles Investigation of Phase Stability, Electronic Structure and Optical Properties of MgZnO Monolayer

**DOI:** 10.3390/ma9110877

**Published:** 2016-10-27

**Authors:** Changlong Tan, Dan Sun, Xiaohua Tian, Yuewu Huang

**Affiliations:** 1 College of Applied Science, Harbin University of Science and Technology, Harbin 150080, China; wyysundan@163.com (D.S.); xiaohuatian@hrbust.edu.cn (X.T.); 2 School of Materials Science and Engineering, Harbin Institute of Technology, Harbin 150001, China; wuyuehuang@gmail.com

**Keywords:** MgZnO monolayer, phase stability, electronic structure, optical property

## Abstract

MgZnO bulk has attracted much attention as candidates for application in optoelectronic devices in the blue and ultraviolet region. However, there has been no reported study regarding two-dimensional MgZnO monolayer in spite of its unique properties due to quantum confinement effect. Here, using density functional theory calculations, we investigated the phase stability, electronic structure and optical properties of Mg*_x_*Zn_1−*x*_O monolayer with Mg concentration *x* range from 0 to 1. Our calculations show that MgZnO monolayer remains the graphene-like structure with various Mg concentrations. The phase segregation occurring in bulk systems has not been observed in the monolayer due to size effect, which is advantageous for application. Moreover, MgZnO monolayer exhibits interesting tuning of electronic structure and optical properties with Mg concentration. The band gap increases with increasing Mg concentration. More interestingly, a direct to indirect band gap transition is observed for MgZnO monolayer when Mg concentration is higher than 75 at %. We also predict that Mg doping leads to a blue shift of the optical absorption peaks. Our results may provide guidance for designing the growth process and potential application of MgZnO monolayer.

## 1. Introduction

Two-dimensional (2D) materials present rather unique and exceptional properties due to the restrictions of size in one dimension and emerging 2D confinement effects. Moreover, they are easier to fabricate into complex structures than in 1D state. The discovery of 2D graphene has led to the intense interest in other 2D materials with novel properties [1]. ZnO displays a direct and wide band gap, large exciton binding energy, and strong piezoelectricity, and is widely applied in blue/UV light-emitting diodes, photocatalysts, sensors, and so on [2,3,4,5]. As the allotrope of superior bulk ZnO, the 2D ZnO sheet logically triggers the scientists’ interest [6,7,8,9,10,11,12,13,14,15,16]. The 2D ZnO sheet was firstly theoretically predicted by Freeman, where Zn and O atoms are arranged in planar sheet like in the hexagonal BN monolayer [6]. Experimentally, Tushce et al. were the first to synthesize two-monolayer-thick ZnO(0001) films on Ag(111) substrate, which provided a direct evidence for the presence of planar ZnO sheets [7]. Very recently, in situ observations of free-standing graphene-like ZnO monolayer has been reported [9]. These provide a platform to design ZnO monolayer-based new functional devices.

Band gap modulation in ZnO monolayer is very important for designing its optoelectronic devices. To date, however, there is few reported study regarding this aspect [16]. For ZnO bulk and thin films, previous studies indicate that modulation of band gap could be realized through the doping of a metal element in ZnO. Among various dopants, Mg can be easily doped in ZnO for its similar radius and electronic shell to Zn atom. Many properties of ZnO can be modulated by doping of Mg. For example, Mg doping can increase the band gap, which is critical to the application in blue and ultraviolet region [17]. Therefore, MgZnO is an important element for the realization of band gap engineering to create barrier layers and quantum wells in ZnO-based optoelectronic devices. Recently, many researchers devoted great efforts to the study of the fundamental physical properties of MgZnO alloys [18,19,20,21]. Many theoretical studies and experimental researches on Mg-doped ZnO have achieved a reasonable understanding of the action of doping on crystalline and structures, as well as their optical and electronic properties [22,23]. Moreover, considerable effort has been devoted for the fabrication and characterization of ZnO/MgZnO multiple quantum wells as well as superlattice [24] by molecular beam expitaxy [25], metal-organic vapor-phase epitaxy [26], and pulsed laser deposition [27]. Up to now, most of the existing research on MgZnO focused on bulk and thin films forms [28,29,30,31,32,33,34,35,36]. To the best of our knowledge, there have not been any previous investigations on the MgZnO monolayer in spite of its unique structure and properties different from their bulk counterpart. Moreover, it is worth noting that in the case of bulk material, the phase segregation between ZnO and MgO was observed for Mg concentration x > 0.36 due to the different crystal structures and large lattice mismatch between ZnO and MgO [37]. Thus, the high Mg content single phase wurtzite MgZnO is difficult to be obtained. ZnO bulk is wurtzite structure while 2D ZnO monolayer exhibits graphene-like structure. Since dimensionality is a known factor deciding structure and properties of material, it is of fundamental interest to investigate whether phase segregation can occur in these 2D materials and further to find their phase stability phenomena, which may be different from those in bulk materials.

Therefore, the present study is dedicated to investigate the phase stability, electronic structure, and optical properties of Mg*_x_*Zn_1−*x*_O (*x* = 0.0625, 0.125, 0.25, 0.5, 0.75, 1) monolayer with Mg concentration *x* range from 0 to 1 by means of first principles calculation. Our results show that different from bulk system, the phase segregation has not been observed in the monolayer due to size effect. Moreover, MgZnO monolayer exhibits interesting tuning of the band gap and optical properties with Mg concentration. Our results may provide guidance for designing the growth process and potential application of MgZnO monolayer.

## 2. Calculational Methods

First-principles calculations are based on the density functional theory and the plane wave method as implemented in the CASTEP code [38]. The interaction between ions and electron is described by ultrasoft pseudopotentials [39]. The cutoff energy for the plane wave basis set is set to 400 eV. The Monkhorst-Pack grid of 4 × 4 × 1 is adopted for the brillouin zone integration. The calculations are carried out with spin-polarization and each system is fully relaxed. In all calculations, the convergence criteria for changes in energy, maximum force, and maximum displacement are 1.0 × 10^−5^ eV/atom, 0.03 eV/Å and 0.001 Å, respectively. The MgZnO monolayer systems are modeled using a periodic boundary condition applied in two directions by the supercell method. Along the direction perpendicular to the MgZnO monolayer, 15 Å of vacuum is included to avoid interaction of periodic images. The models of 4 × 4 supercell for ZnO monolayer with one or two Zn atoms replaced by Mg atoms correspond to 6.25 and 12.5 at % of Mg in MgZnO monolayer, respectively. The models of 2 × 2 supercell for ZnO monolayer with one to four Zn atoms replaced by Mg atoms correspond to 25, 50, 75, and 100 at % of Mg in MgZnO monolayer, respectively. Standard DFT calculation generally underestimates the energy gap of ZnO system. In our previous study [16], we used the DFT+*U* method to overcome the band-gap problem, in which the Hubbard parameter of *U* is added to both Zn 3*d* and O 2*p* orbits (*U*_d,Zn_ = 10 eV and *U*_p,O_ = 7 eV). The calculated band gap of 3.36 eV for ZnO bulk is in good agreement with experimental results [2]. Therefore, taking the same *U* parameters, the electronic structure and optical property of MgZnO monolayer have been calculated in this work.

## 3. Results and Discussion

### 3.1. The Pristine ZnO Monolayer

**Figure 1:**
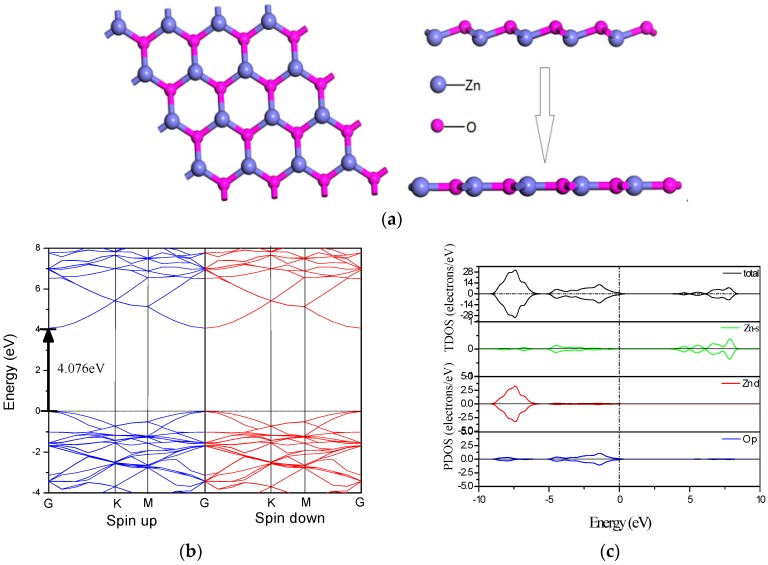
The structural and electronic properties of pristine ZnO monolayer: (**a**) relaxed structure; (**b**) band structure; (**c**) DOS.

To investigate the properties of MgZnO monolayer, the structural and electronic properties of pristine ZnO monolayer are necessary to be calculated. Starting from a structure cut from the ZnO wutrztie crystal and terminated with the (0001) polar surface, atomic relaxations from the DFT calculations indicate that ZnO monolayer stabilizes in a hexagonal BN structure, as shown in Figure 1a. Unlike fourfold coordinate Zn and O atoms in the bulk wurtzite structure, each atom in the monolayer is three-fold coordinated, consistent with earlier theoretical predication. The calculated Zn-O bond length in monolayer ZnO is 1.91 Å, which is lower than the corresponding bond length in bulk ZnO of 2.01 Å. Regarding of electronic structure, the calculated band structure and density of states of ZnO monolayer are shown in Figure 1b,c, respectively. The band structure indicates that the pristine ZnO monolayer is a nonmagnetic semiconductor and retains the direct band gap characteristic of their wurtzite bulk. Our DFT+*U* calculations give a band gap of 4.08 eV, which is more close to the experimental value of 4.50 eV than those obtained in previous DFT calculations [10,40,41]. The improvement for the prediction of band gap of ZnO monolayer in present work than previous studies is attributed to that *U* values are included for both Zn 3*d* and O 2*p* orbits. Moreover, it should be pointed out that the band gap of pristine ZnO monolayer is enlarged with respect to the bulk ZnO band gap (3.37 eV). This phenomenon is confirmed due to quantum confinement effects from both theoretical and experimental aspects [42,43]. Moreover, from density of states (DOS) of pristine ZnO monolayer shown in Figure 1c, it can be seen that the valence bands near the Fermi level are dominated by O 2*p* states and the conduction bands are mainly ascribed to the states of O 2*p* and Zn 4*s* states.

### 3.2. Phase Stability of MgZnO Monolayer

It is generally regarded that in the case of MgZnO bulk material, the phase segregation between ZnO and MgO was observed for Mg concentration *x* > 0.36 due to the different crystal structures and large lattice mismatch. This phase segregation significantly limits the application of MgZnO material. Considering that dimensionality is a known factor deciding structure and properties of material, it is of fundamental interest to investigate if phase segregation can occur in 2D MgZnO monolayer. In order to find their phase stability phenomena, the formation energies (Δ*E*) of MgZnO monolayer with various Mg concentrations are calculated, as shown in Table 1. The formation energy is defined as follows: Δ*E*(*x*) = *E*(Mg*_x_*Zn_1−*x*_O) − {*x* *E*(MgO) + (1 − *x*) *E*(ZnO)}, where *E*(Mg*_x_*Zn_1−*x*_O), *E*(MgO), and *E*(ZnO) are the total energies per supercell of the relaxed monolayer, respectively [44,45]. The negative formation energy means the formation of the monolayer is thermodynamically favorable from the constituents at their stable bulk structures. In terms of all Mg concentration, their formation energies are negative. So we concluded from the formation energies calculations that MgZnO monolayer can possibly be synthesized by the experimental methods, and this MgZnO monolayer should be thermodynamically stable against the phase separation in the corresponding bulk material. For the bulk system, pure ZnO bulk prefers the wurtzite crystal structure, while MgO bulk adopts the cubic rocksalt structure. Therefore, in the case of MgZnO bulk material, the phase segregation between ZnO and MgO was observed due to the different crystal structures and large lattice mismatch. In contrast to bulk system, our formation energies calculations show that MgZnO monolayer including both ZnO and MgO monolayer can keep hexagonal BN structure (graphene-like structure). Indeed, our calculations show that the formation energy of MgO monolayer is far more favorable than other cases. Moreover, the phase stability of MgO monolayer has been verified by other investigations as well [46,47]. Both our studies and previous investigations demonstrate that ZnO monolayer and MgO monolayer exhibit the same hexagonal BN structure. We inferred that this is the internal reason why the phase segregation did not occur in the monolayer. These results demonstrate that 2D ZnO materials exhibit significantly different phase stability phenomena compared to their bulk counterparts and would be useful for designing the growth process and application of MgZnO monolayer.

**Figure 2:**
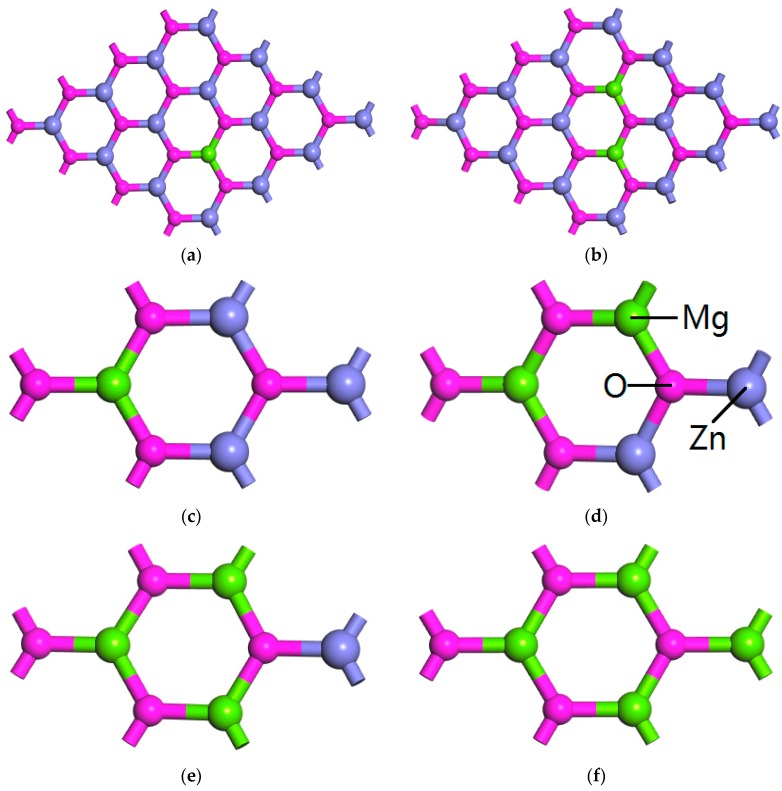
The relaxed structure of Mg*_x_*Zn_1−*x*_O monolayer (**a**) *x* = 0.0625; (**b**) *x* = 0.125; (**c**) *x* = 0.25; (**d**) *x* = 0.5; (**e**) *x* = 0.75; (**f**) *x* = 1.

Furthermore, the structural parameters for MgZnO monolayer with various Mg concentrations after the structural optimizations have been listed in Table 1. The relaxed structures of MgZnO monolayer are shown in Figure 2. It can be seen that the calculated bond lengths between Mg atoms and the nearest-neighbor O atoms are slightly smaller than that of the Zn-O bond length. The calculated Mg-O bond length different from the Zn-O bond length is due to the different ionic radius between Zn and Mg atoms. The O-Mg-O bond angle is approximately 119°. Considering the O-Zn-O bond angle is 120° in the pristine ZnO monolayer, it can be known that Mg doping distorts the bond angle slightly. The above results show that all the lattice structure MgZnO monolayer with different Mg concentration after geometric optimization still keeps graphene-like structure as that of pristine ZnO monolayer.

### 3.3. Electronic Structure of MgZnO Monolayer

**Figure 3:**
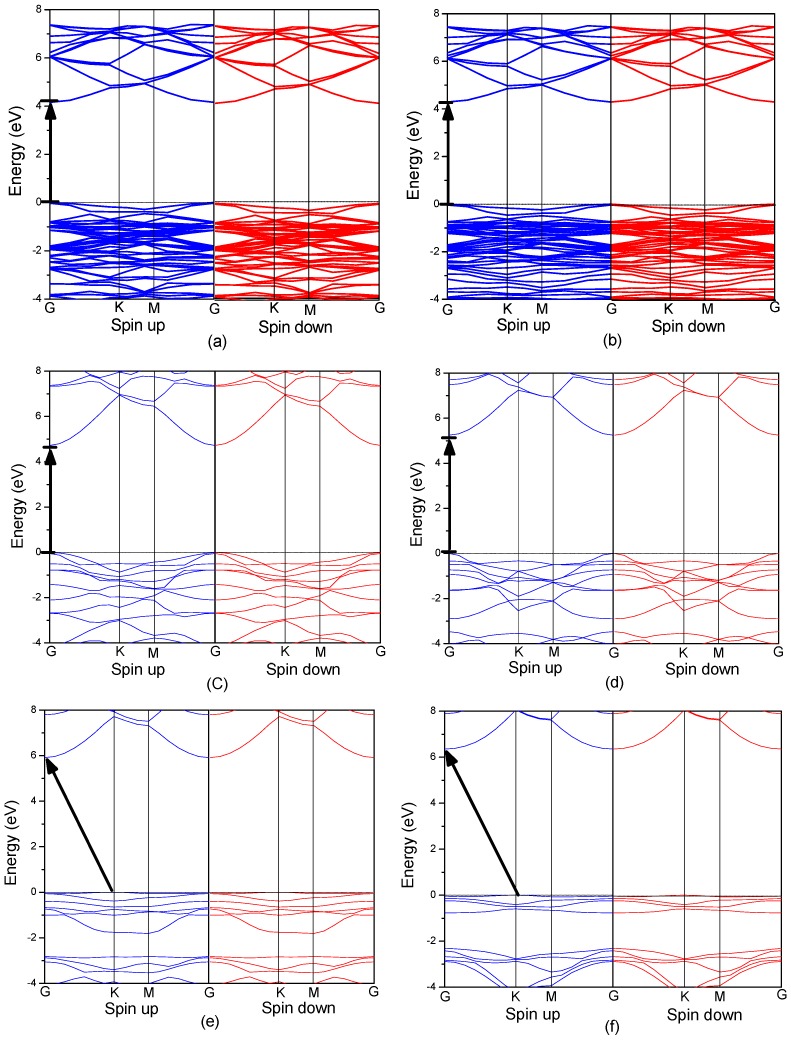
The band structures of Mg*_x_*Zn_1−*x*_O monolayer (**a**) *x* = 0.0625; (**b**) *x* = 0.125; (**c**) *x* = 0.25; (**d**) *x* = 0.5; (**e**) *x* = 0.75; (**f**) *x* = 1.

**Figure 4:**
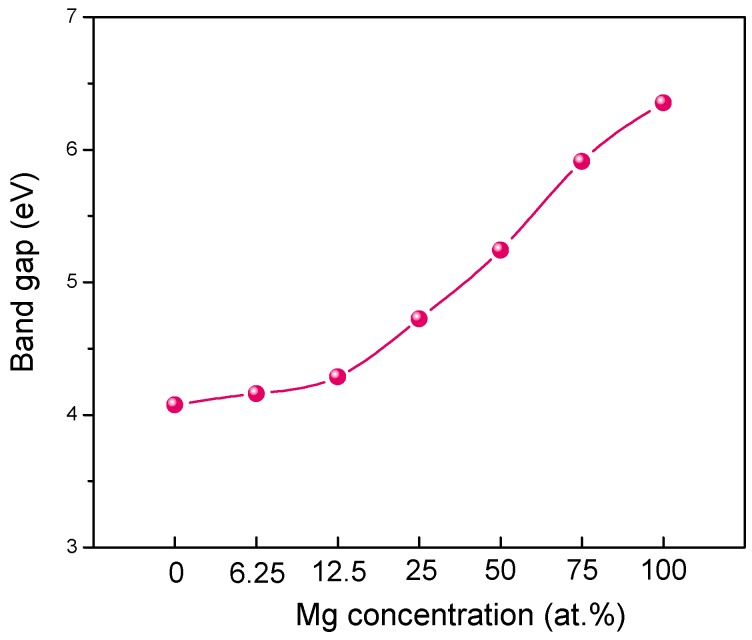
The band gap of MgZnO monolayer versus Mg concentration.

Figure 3 shows the band structure of MgZnO monolayer with various Mg concentrations. It is found that the valence band maximum in MgZnO monolayer is always at the Fermi level and it has no obvious shift with Mg doping. However, the bottom of the conduction band shifts to a higher energy side with increasing Mg concentration. The bottom of the conduction band mainly consists of Zn 4*s* states. When the Mg concentration increases, the Zn 4*s* states at the bottom of conduction band become weaker, resulting in the shift of conduction band and band gap widening. We summarize the results for band gap versus Mg concentration for MgZnO monolayer, as shown in Figure 4. Overall, there is a monotonous increase of band gaps with increasing Mg concentration, which is similar to the previous study of MgZnO bulk. Particularly, a direct to indirect band gap transition is observed for MgZnO monolayer when Mg concentration is higher than 75 at %. In the case of Mg concentration lower than 75 at %, MgZnO monolayer remains a direct band gap semiconductor with both the top of valence band and the bottom of the conduction band located the *G* point, which is similar to that of pristine ZnO monolayer. However, when Mg concentration is higher than 75 at %, the band structure of MgZnO monolayer transforms to indirect band with the valence band maximum (VBM) and the conduction band minimum (CBM) located at *K* and *G* symmetry points, respectively. This study for MgZnO monolayer has provided a simple route to systems in which band gap engineering from a direct to and indirect band gap semiconductors is possible. Moreover, it is well known that the energy levels close to the Fermi level generally play main roles in the electrical and optical properties of materials, so we focus the VBM and CBM. As shown in Figure 3, with increasing Mg concentration, the shape of the bottom conduction band gradually becomes more parabolic (or spherical) with increasing Mg content and the width of the top valence band is narrowed gradually. The shape of bottom conduction band is usually related to the effective mass of electrons. The change of bottom conduction band shape would affect the effective mass of electrons. So the electrical properties of MgZnO monolayer may be tuned by Mg concentration.

**Figure 5:**
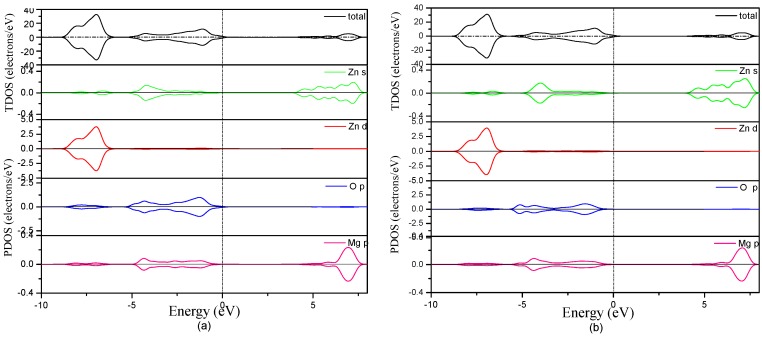

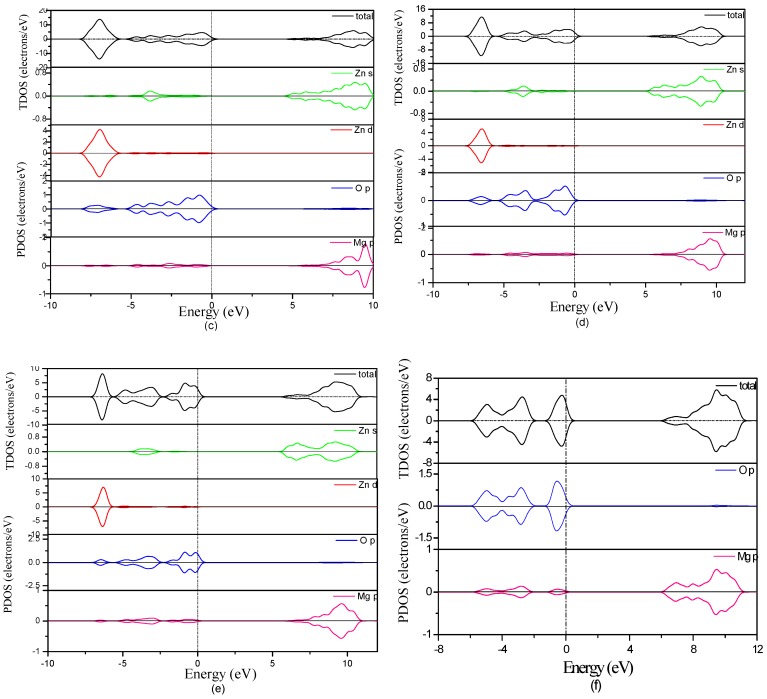
The total and partial DOS of Mg*_x_*Zn_1−*x*_O monolayer (**a**) *x* = 0.0625; (**b**) *x* = 0.125; (**c**) *x* = 0.25; (**d**) *x* = 0.5; (**e**) *x* = 0.75; (**f**) *x* = 1.

The density of states (DOS) is an important part of the electronic property of semiconductor materials. Furthermore, the total DOS (TDOS) and partial DOS (PDOS) of MgZnO monolayer are presented in the Figure 5. It can be seen that the total DOS of MgZnO monolayer is similar to those of pristine ZnO monolayer. The valence bands come mainly from the O 2*p* and Zn 3*d* states, whereas the conduction bands originating from the Zn 4*s* and Mg 2*p* states. It is worth noting that Mg 2*p* states are higher than that of Zn 4*s* states in energy position. With increasing Mg concentration, the CBM determined by Zn 4*s* and Mg 2*p* states shifts to high energy range obviously while the VBM determined by O 2*p* states nearly remains unchanged. This is the main reason for the increase of the band gap in the MgZnO monolayer with increasing Mg concentration. Moreover, the Zn 3*d* states decreased slightly with increasing Mg concentration in the MgZnO monolayer. It is well known that the Mg has no occupied *d* orbit, thus Mg incorporation decrease the hybridization between the Zn 3*d* and O 2*p* states. Moreover, it is found that the O 2*p* and Zn 3*d* states become increasingly localized with a narrower band width with increasing Mg concentrations. The enhanced localization in the O 2*p* and Zn 3*d* states indicates more ionic character in MgZnO monolayer, which also benefits the increase of the band gap.

### 3.4. Optical Properties of MgZnO Monolayer

**Figure 6:**
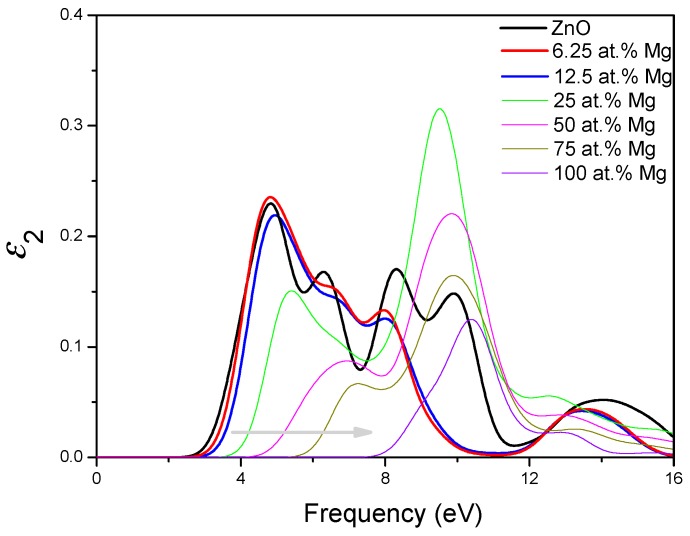
The imaginary part of dielectric function of MgZnO monolayer.

**Figure 7:**
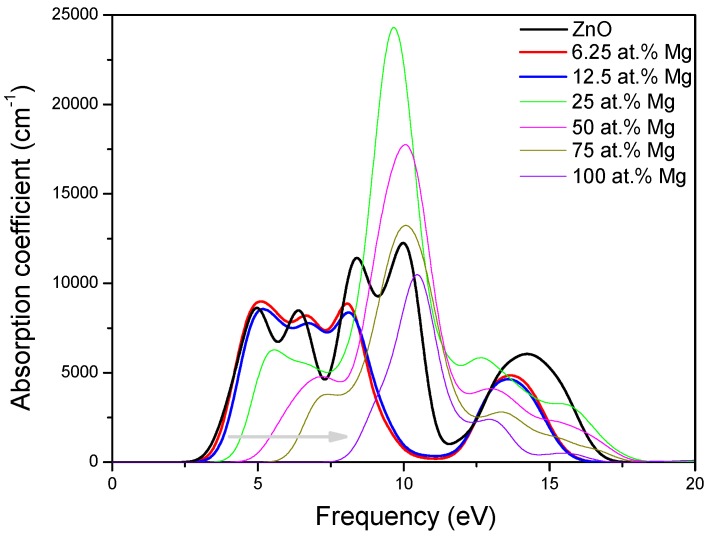
The absorption coefficient of MgZnO monolayer.

In general, the optical properties of semiconductor are closely related to their electronic structures and are of great importance to its application. Thus, we investigate the optical properties of MgZnO monolayer. The imaginary part $\varepsilon_{2}\left(\omega \right)$ of the dielectric function can be calculated from the momentum matrix elements between the occupied and unoccupied wave functions and the real part $\varepsilon_{1}\left(\omega \right)$ of the dielectric function can be evaluated from the imaginary part by the Kramer-Kronig relationship [48]. The absorption coefficient can then be obtained from $\varepsilon_{1}\left(\omega \right)$ and $\varepsilon_{2}\left(\omega \right)$. It is known that the imaginary part $\varepsilon_{2}\left(\omega \right)$ of the dielectric function is very important for any materials, thus for MgZnO monolayer are presented in Figure 6. It can be seen that there are three main peaks in for MgZnO monolayer with different Mg concentrations. In the case of pristine ZnO monolayer, the first peak at 4 eV should mainly be caused by the transition between O 2*p* states in the highest valence band and Zn 4*s* states in the lowest conduction band, which is related to the direct band gap. The second peak at 8 eV can be due to the transition between the Zn 3*d* and O 2*p* states. The weak peak at 14 eV is mainly derived from the transition between the Zn 3*d* and O 2*s* states. The main effect of Mg doping lies in that the first peak due to the optical transition between the Zn 4*s* and O 2*p* states obviously shifts toward the high energy side with increasing Mg concentration, indicating that the band gap is increasing. Moreover, the strength of the first peak obviously decreases with the increase of Mg concentration. This is due to that the increase of Mg concentration corresponds to decrease of Zn 4*s* states, resulting in the decrease of transition between the VBM and CBM. Furthermore, we have calculated the absorption coefficient of pristine ZnO monolayer and MgZnO monolayer, as shown in Figure 7. The changes of line shapes, energy positions, and strengths of the peaks of absorption spectrums with the increasing Mg concentration are similar to those of the imaginary part of the dielectric function. Particularly, it is worth noting that compared to pristine ZnO monolayer, the optical absorption edge of MgZnO monolayer has a clear blue-shift to a shorter wavelength region with increasing Mg concentration, which may have potential application for designing ultraviolet optoelectronic devices. The blue-shift of the absorption edge could be ascribed to the increase of band gap induced by Mg incorporation.

## 4. Conclusions

In conclusion, we report for the first time the first-principles investigations of phase stability, electronic structure, and optical properties of MgZnO monolayer. It is found that the phase segregation occurred in bulk system has not been observed in the monolayer due to size effect. MgZnO monolayer keeps the graphene-like structure with various Mg concentrations. Interestingly, MgZnO monolayer exhibits interesting tuning of electronic structure and optical properties with Mg concentration. The band gap increases with increasing Mg concentration. Particularly, a direct to indirect band gap transition is observed for ZnMgO monolayer when Mg concentration is higher than 75 at %. Furthermore, we also found that Mg doping leads to a blue shift of the optical absorption peaks. These results may provide guidance for the potential application of MgZnO monolayer in ultraviolet optoelectronic devices at nanoscale.

## Figures and Tables

**Table 1:** The structural parameters and formation energies for MgZnO monolayer.

Composition	Bond Length (nm)		Bond Angle (°)		Δ*E* (eV)
	*d_Zn-O_*	*d_Mg-O_*	*θ_O-Zn-O_*	*θ_O-Mg-O_*	
0 at % Mg	0.1910	—	120	—	0
6.25 at % Mg	0.1906	0.1897	119.37	120	−2.7302
12.5 at % Mg	0.1910	0.1906	117.79	118.63	−5.4635
25 at % Mg	0.1911	0.1902	119.37	119.91	−0.6607
50 at % Mg	0.1925	0.1899	119.061	119.19	−1.4450
75 at % Mg	0.1912	0.1912	—	119.59	−2.3109
100 at % Mg	—	0.1881	—	120	0
